# An Analytic Dissection of a Case of Cerebral Diplopia: Is the Human Brain a Holographic Device?

**DOI:** 10.7759/cureus.10292

**Published:** 2020-09-07

**Authors:** Hassan Kesserwani

**Affiliations:** 1 Neurology, Flowers Medical Group, Dothan, USA

**Keywords:** vision disturbance, brain infarct, mathematics

## Abstract

We describe the case of a 70-year-old woman who developed a cerebral infarct of the striate cortex, V1, and associated visual association cortex, V2. She presented with the visual perception disorder of a duplicated image of objects, lower fidelity, and a diaphanous copy of the original (polyopia) that was eerily similar to a hologram. We seize upon this opportunity to explain the generation of these false images. This led us to no less than the spectacular holonomic brain theory, which explains the stupendously high entropy of the brain, the storage of data in the cerebral cortex, the equipotentiality of brain tissue, and the ability of the brain to compute algorithms and perceive sensation in unison. This remarkable ability of the human brain entails the deployment of mathematical Fourier transforms and the electrical slow potentials in the highly interconnected and dense dendritic trees of the cerebral cortex. The ideas explored here are sublime and deep. These machinations are thought to be deeply ingrained in the very fabric of nature; in no less than black holes and the cosmos itself. Our case provides evidence for the holographic model of brain function in a graphic and vivid manner.

## Introduction

Polyopia is a disorder of cerebral function whereby a person perceives an object as duplicated twice or multiple times. The duplicated object is usually fuzzy in appearance and almost ghostly or diaphanous. The duplicated object is usually of the same size as the original and is perceived as unreal, a mere copy. The common denominator in most instances of polyopia is a lesion of the visual association cortex, in the occipital lobe, unilateral or bilateral. By definition, a refractive error and cranial nerve or brainstem ophthalmoplegia are completely excluded. Invariably, there must be occipital lobe dysfunction, which may arise from trauma, ischemic stroke, epilepsy, or even migraine [1-4]. Various theories have been posited to explain the underlying mechanisms of polyopia, including abnormal cortical discharge, a positive phenomenon akin to an aura and hence spreading cortical depression and abnormal visual integration [5]. However, these theories are non-encompassing and somewhat vague and are lacking in mathematical and experimental rigor.

In order to understand the generation of polyopia with the aberrant duplication of multiple images, we need to reconcile many facts; the duplication of images of the same size, the fuzzy and diaphanous quality of the image, their transient nature lasting the duration of fixation, and the fact that the multiplication of images can reach up to 200 copies [5]. We need an underlying mathematical theory that will stitch together all these observations, a theory that is supported by experimental data and one that can take it into account the fact that the human brain can store a vast amount of data, with redundancy and potential for recovery. A striking feature about brains is the fact that most of the data in the brain is stored on the surface, in the cerebral cortex. Can this fit into our new theory? One such theory that has proven to be no less spectacular than any is the phenomenon of holography. The false images, if we are permitted to use this term, have an uncanny resemblance to a hologram. The concept of the hologram has been catapulted to explain how information is stored in the whole cosmos, starting with no less than black holes. The science here is breathtakingly sublime and revolutionary.

The classic hologram is an image that is created when a deflected laser light that struck an object directly interferes with the same afferent light that was reflected from a half-silvered mirror. The new false image is a duplicated image with corrupted fidelity, but nevertheless is a copy with highly similar features [6].

We present the case of a 70-year-old woman who presented with polyopia; the duplication of an image upon fixation at a distance. This false image was a fuzzy diaphanous duplicate of the same size. It was transitory. The most proximate etiology was an ischemic infarct of the visual striate cortex, V1, and the nearby visual association cortex, V2. In the discussion, we explain how a complex, high entropy information storage and programmable system like the human brain can generate such duplicate images by the holographic (holonomic) brain theory. We believe that the characteristic features of the duplicated image of this case fit with a hologram. The holonomic theory explains how the brain is able to store a fantastic sum of data, memory recall, and the equipotentiality of brain tissue, that is, Karl Lashley's mass action; the ability of remaining brain tissue to compensate for lost tissue [7]. We will briefly discuss, in layman terms, the mathematical basis and experimental data of this radical theory. This entails a brief understanding of Fourier transforms and some basic ideas in diffraction optics.

## Case presentation

**Figure 1:**
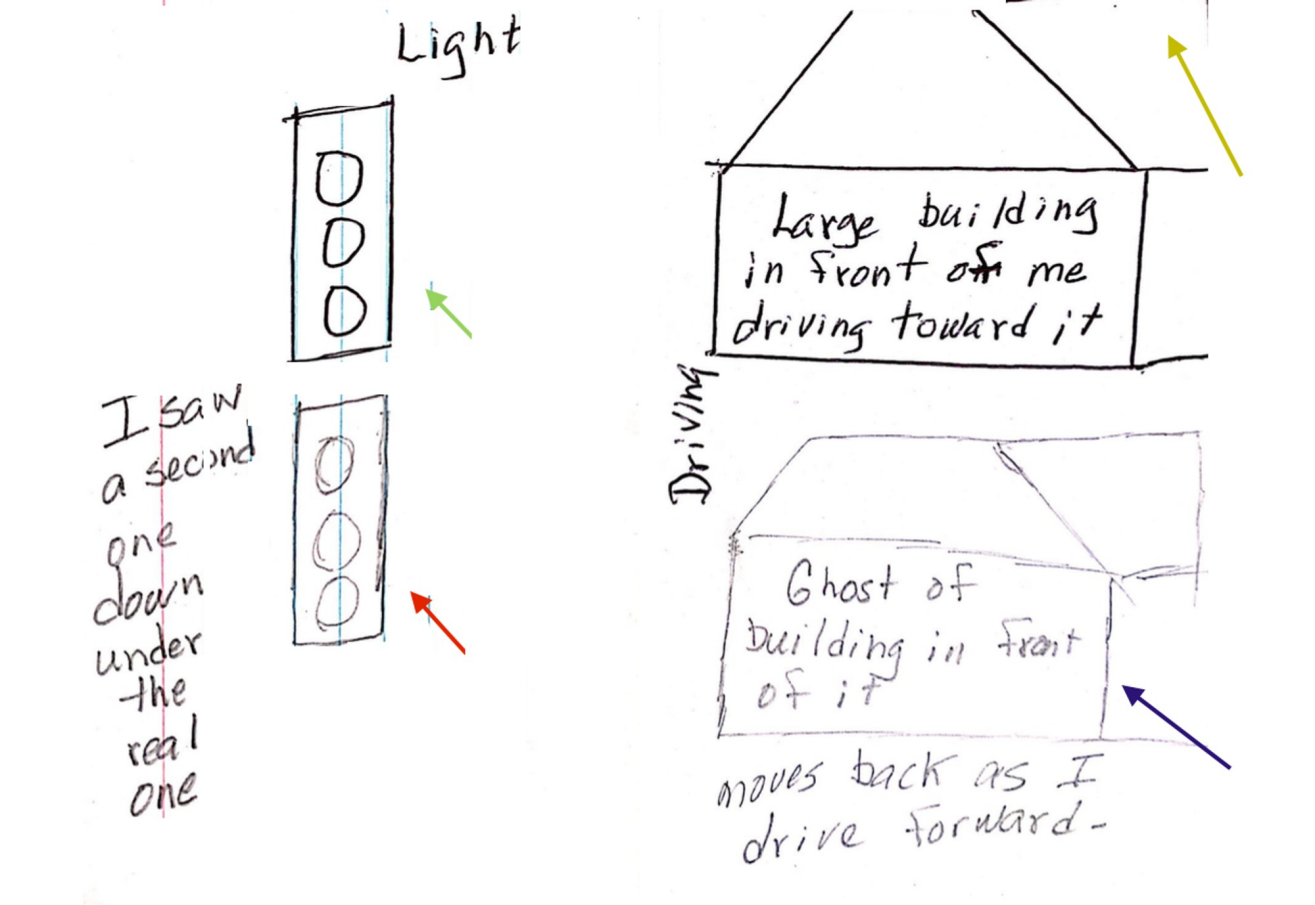
Drawing by the patient The patient's own original rendition scribbled on a random piece of paper: duplicated traffic light (red arrow) of the same size as the original (green arrow). Duplicated house (blue arrow) recedes as she drives towards it. The lines in the top right-hand corner are part of the duplicated image and not an artifact (yellow arrow).

We describe the case of a 70-year-old woman who developed sudden-onset vertical separation of images. This occurred only when she specifically gazed at a distance. For example, when she looked at a plant, she saw a double image that was completely vertically separated, without overlap. The bottom image was blurry and with color but of a similar size. It appeared ghostly and almost transparent. She could almost see through it; it was diaphanous. However, it did not disappear with either eye closed. The blurry second-bottom image disappeared only with the closure of both eyes or by looking away from the object. Hence, there was no evidence of palinopsia or bona fide diplopia. This phenomenon occurred for both stationary and moving objects. A moving truck was experienced as two vertically separated images, with the bottom image described as blurry. She knew that the bottom duplicated image was not real. The duplicated image was of the same size and receded as she approached it, as demonstrated by the patient's own original rendition (Figure 1).

She denied any visual field loss or scotoma. The color perception was preserved. She denied photopsia, micropsia, macropsia, or metamorphopsia. Visual acuity was not diminished, and she was still able to read and write with ease. She denied a headache or weakness of the arms or legs. She saw an ophthalmologist who noted full ocular motion without any subtle ophthalmoparesis on double Maddox rod testing.

Her past medical history was significant for asthma and ulcerative colitis. Her medications included azathioprine, lansoprazole, mesalamine, and montelukast. Her mom had coronary artery disease and her dad had cerebrovascular disease. She was a non-smoker.

On examination, her blood pressure (BP) was 150/92 with a pulse of 71, which was regular. Her height is five feet with a weight of 185 pounds and a body mass index (BMI) of 36.1. The precordial examination was negative for a murmur and carotid bruits were absent in both internal carotid arteries. Her neurologic examination was non-revealing. Her gait station, cadence, and tandem walking were normal. Her cranial nerve examination was entirely normal. Specifically, the ocular motion was full without strabismus with a cover/uncover test and an alternate eye cover test. Accommodation was preserved without pupillary asymmetry, and visual field confrontation was normal. We were careful not to miss visual field neglect by sequential hemifield visual confrontation followed by simultaneous double hemifield confrontation. The rest of the cranial nerve function was normal. Visuomotor praxis was preserved with pantomime. No limb-kinetic or ideomotor apraxia was noted with coin deftness and transitive motor acts respectively. Ishihara plate color testing was normal. Power function was normal throughout and graded at 5/5 with the Medical Research Council (MRC) grading. Sensory examination was normal to graphesthesia and stereognosis in both hands. And deep tendon reflexes were symmetric and lively in the arms and legs. No dysmetria or intention tremor was noted in the arms. Heel to shin motion was normal in the legs bilaterally.

**Figure 2:**
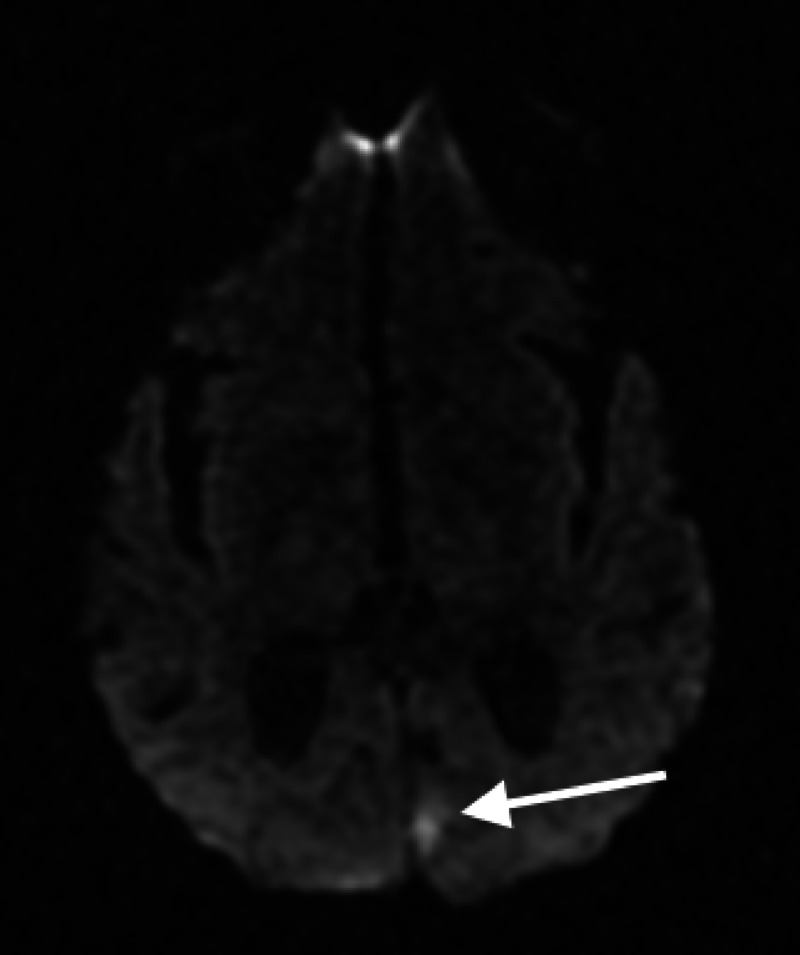
MRI: (DWI) demonstrating ischemic infarct of striate cortex, V1, and associated visual association cortex, V2 (white arrow) MRI: Magnetic Resonance Image; DWI: Diffusion-Weighted Imaging

A magnetic resonance image (MRI) of the brain shows a diffusion-weighted image (DWI) hyperintensity involving the left mesial occipital lobe; striate cortex, V1, spilling over into the adjacent visual association cortex, V2, indicating an ischemic infarct (Figure 2).

**Figure 3:**
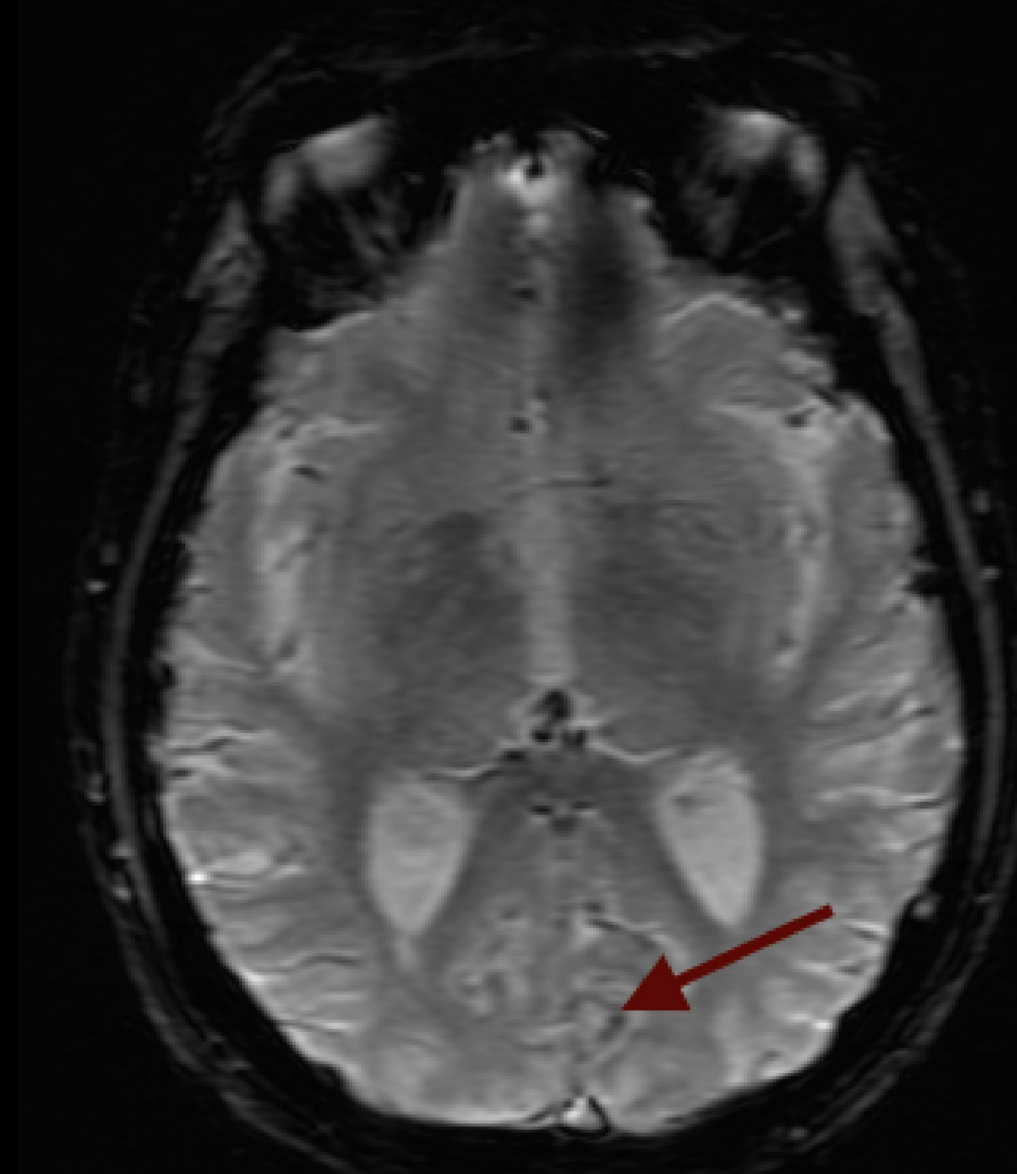
MRI: GRE demonstrating corresponding the hypointensity of the visual association cortex, V2 (red arrow) MRI: Magnetic Resonance Image; GRE: Gradient Echo Image

There was a corresponding hypointensity on gradient echo imaging (GRE) in the region of V2 (Figure 3).

**Figure 4:**
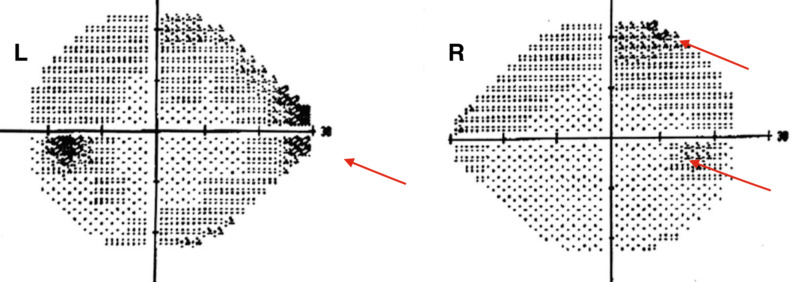
Humphrey automated visual field test Subtle and patchy mild incongruous right homonymous hemianopia (red arrows): left eye (L), right eye (R)

A carotid duplex scan was normal. A transcranial Doppler (TCD) with a two Megahertz crystal with the insonation of temporal, transorbital, and sub-occipital windows bilaterally showed monophasic waveforms with no high-velocity jets, which was normal. Due to the embolic nature of the ischemic infarct and a normal event monitor, a cardiac loop monitor was implanted to rule out paroxysmal atrial fibrillation. A transthoracic echocardiogram with a bubble study did not reveal a patent foramen ovale or any evidence of cardiac vegetations or intra-cavitary thrombus. The automated Humphrey visual field test showed a subtle and patchy incongruous right homonymous hemianopia (Figure 4); the patient herself did not notice any visual field deficits.

The patient was defaulted to 81 milligrams (mg) of aspirin daily.

## Discussion

We will outline the concept of the Fourier Transform (FT), the concept of the brain as a parallel holistic processor, detail the basics of the dense cortical dendritic web, and how it relates to holography. Finally, we stitch together these concepts in order to understand the holographic (holonomic) nature of visual processing. We end by relating these deep and complex ideas to our patient's polyopia. We will henceforth refer to the duplication of images as polyopia, the simplest duplication being diplopia.

**Figure 5:**
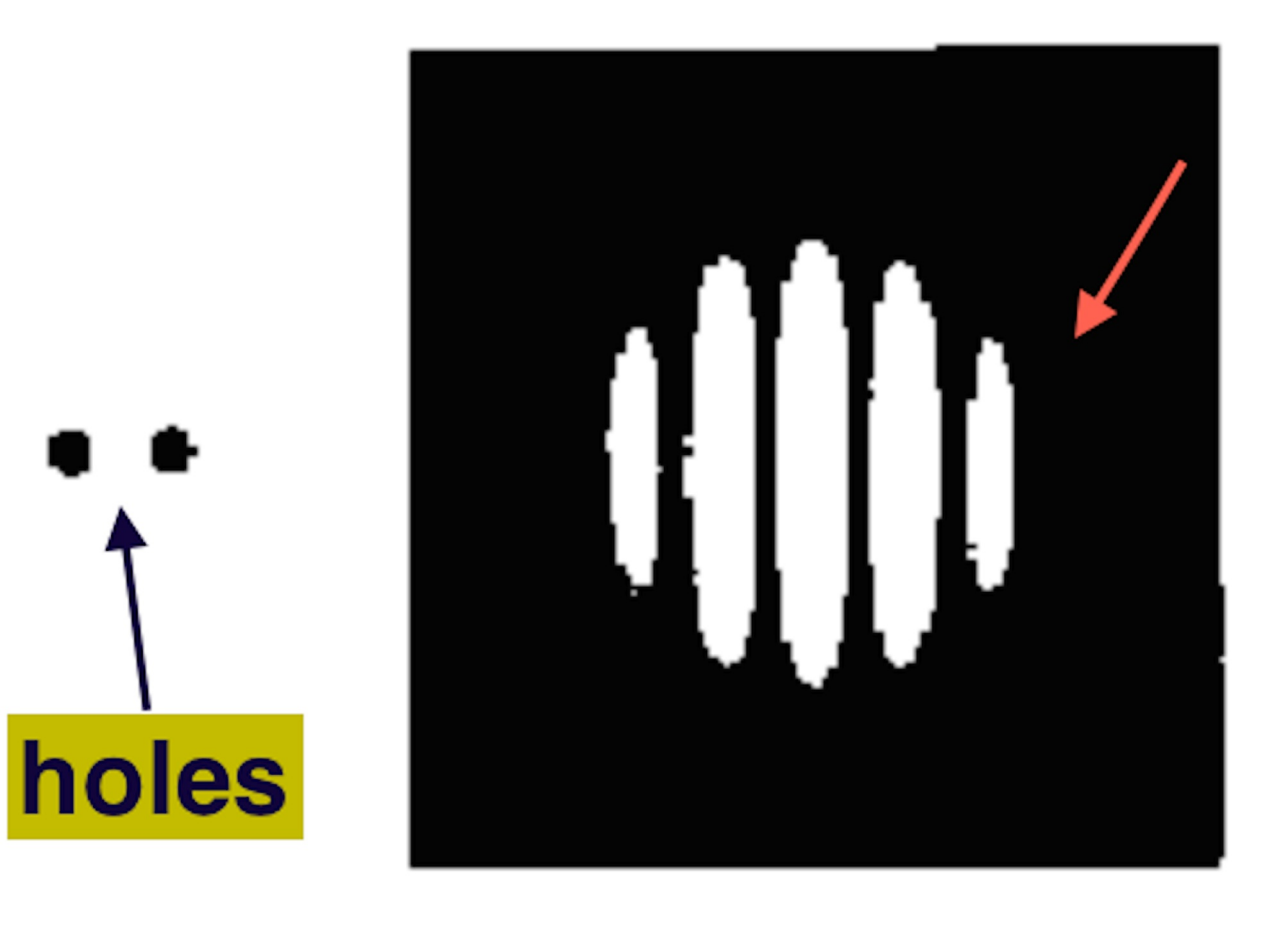
Fourier Transform of light incident upon two holes The diffraction pattern (red arrow) is the Fourier Transform of light entering both holes (black arrow).

FT is at the heart of modern technology and nature. It is deployed everywhere; image processing by the lens, retina and visual cortex, modern computer image processing, power spectra of sound and music, in jpeg file compression, speech recognition and hearing aids, magnetic resonance technology, and many more applications. The magic of the mathematical technique of FT is its ability to extract data and flip flop between two variables [8]. FT comes in pairs: frequency function and time function in signal analysis, displacement function, and momentum function in quantum mechanics, and aperture length and sine function of angle of diffraction in diffraction gratings. The latter is what concerns us here because diffraction and interference is natures' way of computing FT. That is, light passing through a slit under the right conditions, such as narrow bandwidth and far-field, will produce the FT of the slit or aperture (Figure 5).

The essence of FT in data extraction is to deconstruct a complex wave function and produce a power spectrum. The power spectrum is a visual representation of the main frequency bands contributing to the signal. It is a method of deconstruction and construction; we can travel in either direction. For our purposes, we are interested in the FT pair of spatial frequency and time-frequency domain as it pertains to brain function. These are not mutually exclusive in the hemispheres or anterior and posterior lobes of the brain. However, there are interesting patterns that arise in the brain, with the right hemisphere being holistic and synthesizing images in a Gestalt pattern and the left hemisphere behaving algorithmically and analytically [9]. The essential features of the holistic and analytic approaches to brain function are compared (Table 1).

**Table 1:** Comparing the holistic and analytic mode of function of both hemispheres: these modes are not mutually exclusive and both can exist in the same hemisphere

HOLISTIC	ANALYTIC
Visual system and light	Auditory system and sound
Right hemisphere - postero-lateral - somatotopic and visual input	Left hemisphere - limbic, auditory, and olfactory
Image mode	Processing and programming mode
Esthetic quality	Either works or not: if faster, better
Gestalt principles	Mechanistic - transistorized

There is intermixing between the two modes of signal transduction in both halves of the brain, and this comparison is not mutually exclusive.

We postulate that the corpus callosum may act as a Fourier transformer between both hemispheres. If the right occipital cortex acts in the frequency domain, which we know is true, and hence can extract spatial information, it is conceivable that the contralateral visual cortex may process information in the time domain. This is a Fourier pair. By Kantian philosophy, time and space perception is the generator of consciousness. Hence, we have the ingredients of consciousness. Clearly, this needs to be corroborated with functional MRI studies, diffusion tensor imaging, connectome imaging, and intracellular recordings.

In order to understand the holographic theory of the brain, we need to outline certain prerequisites of computing systems. Van Neumann posited in his famous book, The Computer and Brain, that computers are primarily serial and analytic processors [10]. One event leads to another. But the brain is a parallel and holistic processor and hence many related events occur simultaneously. In the human brain, the right hemisphere works predominantly in image mode (perception) and the left hemisphere with program processing. The slow potentials of pre- and postsynaptic potential in the dense horizontal dendritic trees, as described below, produce an interference pattern, which is a hologram. The small wavelength of light in the retina will allow the storage of a fantastic amount of data, which is widely distributed. This is why lesions in the brain may leave no deficits in many locations [11].

Hubel and Weisel in 1959 had shown that visual cortical cells responded to bars and edges of a certain direction [12]. However, in a remarkable and ground-breaking paper in 1971, De Valois showed that recordings in striate cortex neurons in cats and monkeys are spatial frequency filters [13]. Incredibly, different cells that respond to the same patch of the visual field are tuned to different spatial frequencies and orientation. This means that they encode the Fourier spectrum of a patch of visual space. They showed that if a cortical cell were acting as an edge detector, the cells would react optimally by discharging maximally when presented with square-wave gratings, plaids, or checkerboards that are all in the same direction. However, it turns out that the cortical cells respond maximally to a checkerboard pattern that is rotated 45 degrees to both the square wave grating and plaid grating. This is what is expected when the cells are responding to a Fourier fundamental frequency and not as an edge detector. The cortical cells also responded differently to checkerboards of different sizes, and this data fitted with the mathematical predictions. Even more convincing is the fact that cortical cells are more sensitive to the higher harmonics of the Fourier spectrum [12].

The distal end of an axon splits into branches called teledendrons, which connect to other dendrites through electrical ephapses and chemical synapses. The teledendrons and dendrites form a web of fine interconnected fibers. Here, nerve impulses do not occur. Instead, we find depolarizations and hyperpolarizations of electric potential differences, oscillating polarization, in the fine intricate web. The density of synapses and ephapses in this synapto-dendrodendritic web is the highest in human cortices. The frequencies of oscillations of membrane hyperpolarizations and depolarizations in different regions intersect with another, producing patterns of interference in this processing web. The recording of the receptive fields of these neurons is known as holonomy. Information processing takes place in this interconnected web, in what is known as the phase space. The dendrites of neurons sample the phase space to create cell assemblies. The assembly is kaleidoscopic, meaning that each neuron can partake in multiple assemblies. In this way, information is distributed in a holistic, or more aptly, wholistic way. The whole is greater than the sum of its parts and every part is distributed over the whole. Therefore, every part contains the whole. This is the essence of a hologram [11]. In a nutshell, the cerebral cortex like the retina does not register an electric current with intracellular recordings. Instead, they register hyperpolarizations and depolarizations at the dendritic end-plates. These slow potentials interfere with each other and this pattern of interference is a Fourier transform, just as in a diffraction grating. This is the foundation of an engram or a hologram [9].

How do visual cells extract visual information? The Gabor transform is a short-time Fourier transform that is used to determine the frequency and phase content of a short segment of a signal. The Fourier function to be transformed is multiplied by a Gaussian function, a window function, and the result is then transformed with a Fourier transform to derive the time-frequency analysis. The Gabor wavelet transform can extract both time (spatial) and frequency information from a signal, and the tunable kernel size allows it to perform a multi-resolution time-frequency analysis. A smaller kernel size, in the time domain, has a higher resolution in the time domain but a lower resolution in the frequency domain and is used for higher frequency analysis while bigger kernel size has a higher resolution in the frequency domain but a lower resolution in the time domain and is used for lower frequency analysis. This property of the Gabor transform is known as the Gabor uncertainty principle. The wavelet transform is suitable for image compression, edge detection, and object recognition. The cells of the visual cortex of mammalian brains are best modeled as a family of self-similar two-dimensional Gabor wavelets [14].

Our patient developed an ischemic infarct in the left visual cortex areas, V1 and V2, Her duplicated image has all the features of a hologram; exact copy with similar dimensions, diaphanous, transitory (only with fixation at a far distance object, consistent with diffraction and holography) and an isolated phenomenon (without any dizziness or depth perception deficits). To use her own words, ghostly. The patient did not demonstrate a refractive error or cranial nerve dysfunction. Whatever theory we propose has to incorporate all the features of this phenomenon of polyopia. In the holonomic brain theory, we have a solid mathematical foundation with experimental and clinical data. Whereas association does not imply causation, we have attempted to explain this phenomenon in a brief but self-consistent way. This is an area open for critique and is a prime area of research, as it is at the root of a very complex and intangible field; the field of perception and consciousness. We can safely say that the human brain is a three-pound universe, as the table below attests to the wonder of the organ we call the human brain (Table 2).

**Table 2:** Highlighting the power of the human brain A bit can be defined as a unit of information equivalent to the logarithm of 2, base e; where e is the Napierian number

SYSTEM	NUMBER OF BITS OF INFORMATION
HUMAN BRAIN	10 to the power of 69.
BLACK HOLE THE SIZE OF PLANET EARTH	10 to the power of 69
COSMOS	10 to the power of 122

The holographic or holonomic theory of brain function provides a template that explains the duplication of images with disordered brain function, namely, occipital lobe dysfunction; explains the enormous information-carrying capacity of the human brain; and allows for redundancy of function as observed clinically. Furthermore, as outlined above, it has both a mathematical and experimental basis. It is an intriguing theory that has parallel postulates in physics, the holographic theory of black holes, and the cosmos [15]. Naturally, a lot more work needs to be done. But, as clinicians, we are able to observe patients in vivo and accumulate supporting clinical evidence.

## Conclusions

We graphically presented a case of polyopia secondary to an ischemic infarct of the left occipital lobe. We showed that the features of image duplication are eerily similar to a hologram. We presented, in a succinct fashion, the ingredients of the holonomic brain theory, including its mathematics in layman's terms. What makes this theory so attractive is that it has a solid and predictive mathematical and experimental basis. This topic is also a frequently discussed item in the wider scientific community, as the holographic theory is the leading contender to explain information in black holes and the wider cosmos, as was alluded to earlier in the discussion segment. This is inherently a highly complicated topic of great complexity, as it also lies at the heart of consciousness. This article also highlights the power of case reports and their uncanny ability to help us understand the machinery of the human brain and mind.
